# Genotypic and phenotypic characterization of glucose-6-phosphate dehydrogenase (G6PD) deficiency in Guangzhou, China

**DOI:** 10.1186/s40246-023-00473-9

**Published:** 2023-03-22

**Authors:** Ziyan Li, Zhenyi Huang, Yanxia Liu, Yunshan Cao, Yating Li, Yanping Fang, Meiying Huang, Zixi Liu, Lijuan Lin, Lingxiao Jiang

**Affiliations:** Department of Laboratory Medicine, Zhujiang Hospital, Southern Medical University, Guangzhou, Guangdong China

**Keywords:** G6PD deficiency, Genotype, China, Heterozygote

## Abstract

**Background:**

G6PD deficiency is a common inherited disorder worldwide and has a higher incidence rate in southern China. Many variants of G6PD result from point mutations in the G6PD gene, leading to decreased enzyme activity. This study aimed to analyse the genotypic and phenotypic characteristics of G6PD deficiency in Guangzhou, China.

**Methods:**

In this study, a total of 20,208 unrelated participants were screened from 2020 to 2022. G6PD deficiency was further analysed by quantitative enzymatic assay and G6PD mutation analysis. The unidentified genotype of the participants was further ascertained by direct DNA sequencing.

**Results:**

A total of 12 G6PD mutations were identified. Canton (*c.1376G*>*T*) and Kaiping (*c.1388G*>*A*) were the most common variants, and different mutations led to varying levels of G6PD enzyme activity. Comparing the enzyme activities of the 6 missense mutations between the sexes, we found significant differences (*P* < *0.05*) in the enzyme activities of both male hemizygotes and female heterozygotes. Two previously unreported mutations (*c.1438A*>*T* and *c.946G*>*A*) were identified.

**Conclusions:**

This study provided detailed genotypes of G6PD deficiency in Guangzhou, which could be valuable for diagnosing and researching G6PD deficiency in this area.

## Background

G6PD deficiency is one of the most common enzymopathies, affecting approximately 400 million people worldwide [1]. G6PD is a housekeeping enzyme that catalyses the first and rate-limiting step in the pentose phosphate pathway. G6PD provides reducing power in the cytoplasm in the form of NADPH. This process is particularly important in red blood cells, where NADPH serves as an electron donor for detoxifying hydrogen peroxide via reduced glutathione. G6PD is crucial for protecting cells from oxidative stress [2]. Clinical manifestations of G6PD deficiency vary from chronic nonspherical haemolytic anaemia (CNSHA) and acute haemolytic anaemia to severe bilirubin encephalopathy and even death. G6PD deficiency is caused by inherited mutations of the X-linked gene G6PD. The G6PD gene is located in the q28 region of the human X chromosome, with a total length of 18.5 Kb, and contains 13 exons and 12 introns with a total of 515 amino acids. More than 200 mutations of the G6PD gene have been reported worldwide [1], and the distribution of mutation sites varies from country to region.

The first classification of G6PD-deficient variants was made in 1966 [3] and updated by a World Health Organization (WHO) Working Group in 1985 [4]. The classification is still in use today. In 2013, Domingo’s group first proposed a way to use the adjusted male median (AMM) to define the 100% enzyme activity. However, there is no clear AMM value for China, so we need to calculate the AMM value of the local region to define its 100% enzyme activity. In 2022, a new WHO classification of G6PD variants in homozygous and hemizygous individuals was proposed at the Malaria Policy Advisory Group Meeting [5]. The proposed new classification system will include four classes: A (< 20% of G6PD activity/chronic hemolysis), B (< 45% of G6PD activity/acute, triggered hemolysis), C (60–150% of G6PD activity/no hemolysis), and U (any G6PD activity/uncertain clinical significance).

G6PD deficiency is relatively common in historically malaria-endemic countries [6]. The use of antimalarial drugs such as 8-aminoquinoline can cause severe haemolysis in G6PD-deficient individuals. Thus, determining the prevalence of G6PD deficiency in malaria-endemic countries is critical for malaria control and elimination programmes. In the past, malaria was endemic in China. As of today, China has not reported any indigenous malaria cases since 2017 [7] and was certified malaria-free by the WHO on 30 June 2021 [8]. However, some oxidizing drugs and traditional Chinese medicines, such as berberine and honeysuckle, cause a risk of haemolysis in G6PD-deficient individuals in China. Moreover, the detection of G6PD deficiency by enzyme activity measurement may miss individuals with normal phenotypes but positive genotypes. On the other hand, some researchers found that G6PD activity in thalassaemic patients was higher than that in healthy individuals [9]. Thalassemia has been reported to be highly prevalent in southern China [10]. The increase in G6PD activity in patients with thalassemia is closely related to new red blood cells in the body. Haemolysis in thalassemia patients stimulates the body to produce a large number of red blood cells. The G6PD activity in new red blood cells is relatively high [1], which may also lead to missed diagnosis. Therefore, it is also essential to test for G6PD genotypes and understand the relationship between G6PD enzyme activity and genotypes.

Epidemiologically, G6PD deficiency in China is more common in the southern population, and different point mutations lead to G6PD polymorphisms [11, 12]. Guangzhou is a first-tier city in South China with a population of over 20 million. This study aimed to calculate the AMM value for the local region to define its 100% enzyme activity, further detect G6PD mutations and potential pathogenic new mutations in the Guangzhou population, determine the genotypes and distribution of G6PD deficiency, and explore the relationship between enzyme activity and genotypes.

## Materials and methods

### Population

**Fig. 1:**
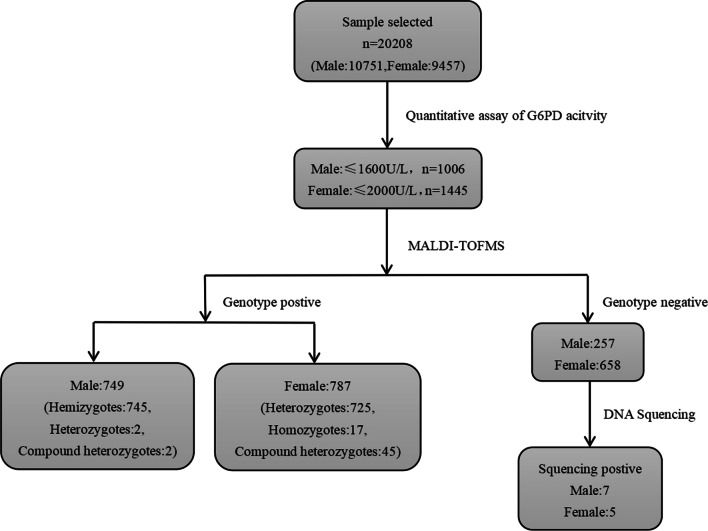
Flowchart of participant enrolment and study design

A total of 20,208 unrelated participants were enrolled in our study from July 2020 to January 2022, including 10,751 males and 9,457 females. Their ages ranged from 14 to 95 years old. This study was a single-centre study. All participants were had undergone physical examination at the centre and agreed to undergo haematological tests. We collected the remaining blood samples from these participants for quantitative assays of G6PD activity, G6PD genotype determination, and gene sequencing. The study protocols were approved by the Ethical Committee of the Zhujiang Hospital of Southern Medical University. The study design is depicted in a flowchart (Fig. 1).

### G6PD enzyme activity measurement

Enzyme activity was measured using a commercial G6PD Detection Assay Kit (Antu Co. Ltd, Beijing, China), the principle of which is to measure the change in the absorbance of NADPH at 340 nm at a given temperature to calculate the activity value of G6PD (U/L). The reference range of G6PD activity provided by the manufacturer is 1300–3600 U/L. All of the tests were conducted by following the manufacturer’s instructions. The reliability of the test results was monitored by calibration and using controls provided by Antu in each test run.

### G6PD genotyping

**Fig. 2:**
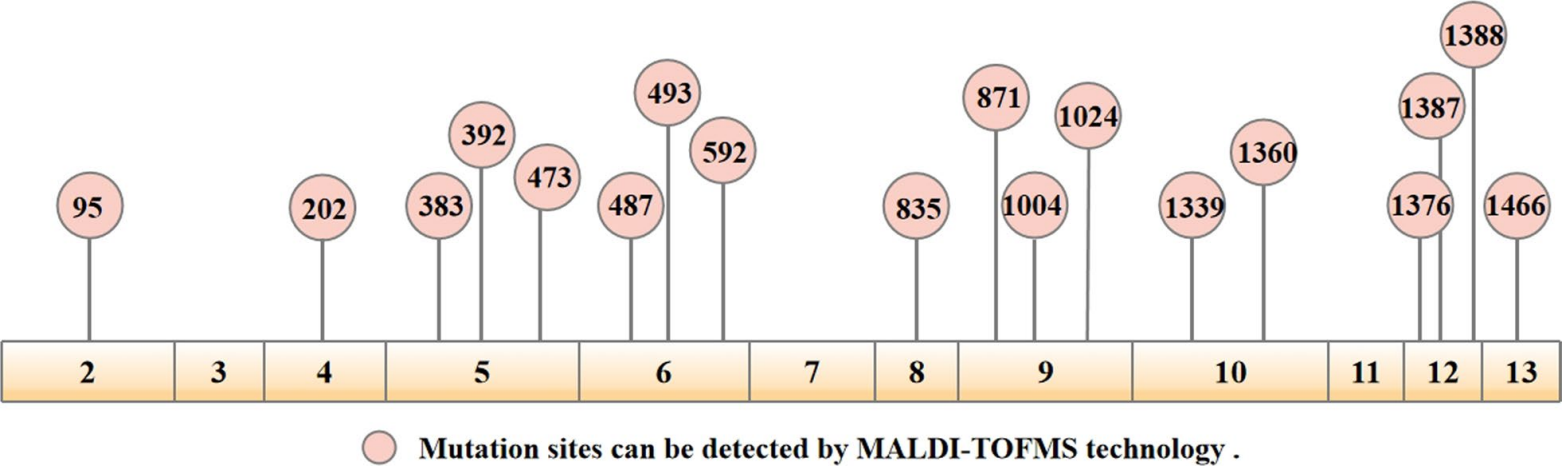
MALDI-TOF MS identified 18 G6PD mutations

Genomic DNA was extracted from peripheral venous blood using a DNA extraction system and kit (Tianlong, Co., Ltd, Xian, China) according to the manufacturer's instructions. Mutations were identified through a method based on multiplex polymerase chain reaction (MPCR) technology and matrix-assisted laser desorption ionization time of flight mass spectrometry (MALDI-TOFMS) technology (Darui Biotechnology Co., Ltd, Guangzhou, China) according to the manufacturer’s instructions. This kit could be used to qualitatively detect the 18 common G6PD mutations in human peripheral blood genomic DNA in China: *c.95A*>*G, c.202G*>*A, c.473G*>*A, c.383T*>*C, c.392G*>*T, c.487G*>*A, c.493A*>*G, c.592C*>*T, c.835A*>*G, c.871G*>*A, c.1004C*>*A, c.1024C*>*T, c.1339G*>*A, c.1360C*>*T, c.1376G*>*T, c.1387C*>*T, c.1388G*>*A* and *c.1466C*>*T* (Fig. 2).

### DNA sequencing

Genomic DNA was extracted from peripheral venous blood by a DNA extraction system and kit (Tianlong, Co., Ltd, Xian, China) according to the manufacturer's instructions. Direct polymerase chain reaction (PCR) sequencing of the G6PD gene in the genomic DNA was carried out. The primers were designed to amplify exons 2–12 of the G6PD gene covering all the coding regions of G6PD.

### Data analysis

All data were analysed by using the Statistical Package for Social Sciences (Released 2013. IBM SPSS Statistics for Windows, Version 22.0. Armonk, NY: IBM Corp) and plotted by Origin 2021 software. The Wilcoxon rank sum test was used for comparisons between patients, as the above results presented a nonnormal distribution. Differences for which P values were < 0.05 were considered to be statistically significant.

## Results

### Determination of G6PD enzyme activity

**Fig. 3:**
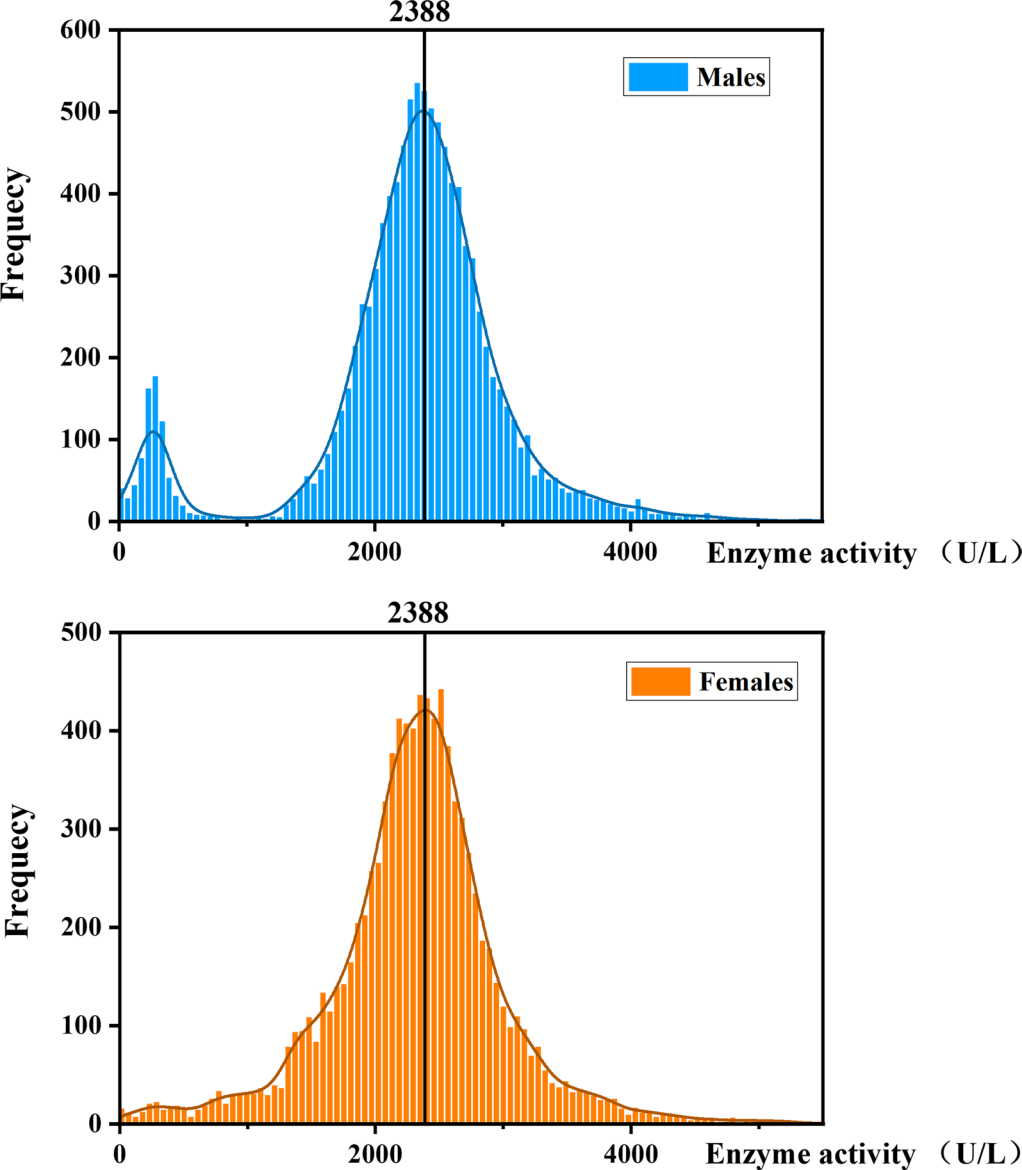
Histogram of G6PD enzyme activity distribution in females and males. The line in the figure represents 100% of the AMM (2388 U/L)

The G6PD enzyme activity distribution in females and males is depicted in the histogram (Fig. 3). The distribution of G6PD enzyme activity in males showed a bimodal and abnormal distribution. The mean ± standard deviation (SD) and median G6PD activities were 2294.98 ± 771.88 U/L and 2379.00 U/L in males. The histogram of the distribution of enzyme activity in females showed a unimodal and abnormal distribution, and the mean ± SD and median G6PD activities were 2328.78 ± 693.88 U/L and 2354.00 U/L. The difference in enzyme activity between males and females was statistically significant (*P < 0.05*).

The AMM is accomplished by: 1. Exclusion of all males with G6PD activity equal to or less than 10% of the male median. 2. Determination of a new median G6PD activity [13]. We calculated the AMM value to define 100% G6PD enzyme activity for this population. In this study, the AMM value was 2388 U/L. Therefore, according to the WHO classification, WHO Class II enzyme activity was < 238.8 U/L (G6PD activity < 10%), and WHO Class III enzyme activity was 238.8–1432.4 U/L (G6PD activity of 10%-60%). We also calculated the 30% AMM value (716.4 U/L), which is as an acceptable level of G6PD activity for primaquine administration [13, 14].

### G6PD genotyping

**Fig. 4:**
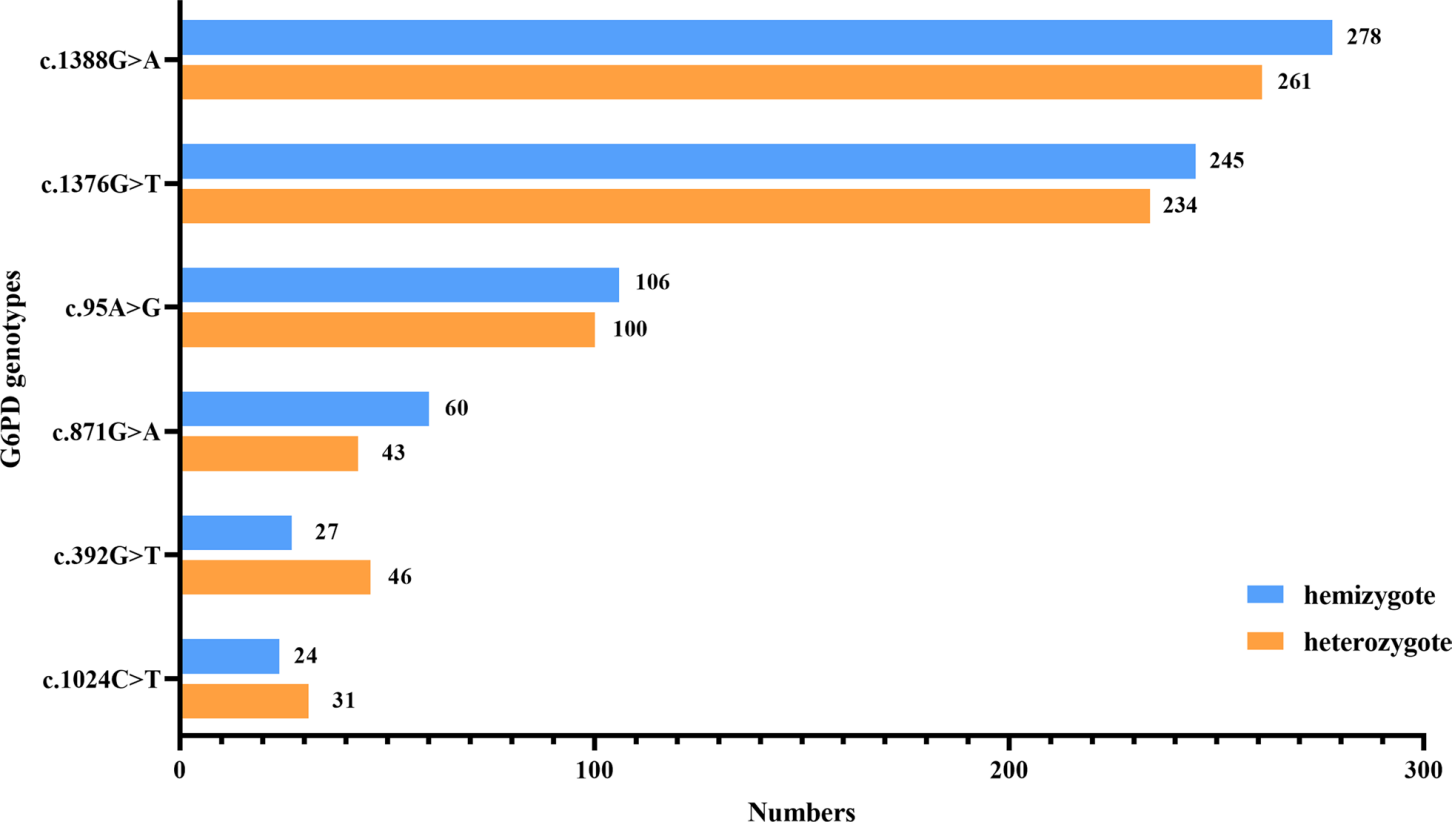
Histogram of males hemizygous and females heterozygous for the six common mutations detected

The results of MALDI-TOF MS showed 12 G6PD mutations, including *c.1376G*>*T*, *c.1388G*>*A*, *c.95A*>*G*, *c.871G*>*A*, *c.392G*>*T*, *c.1024C*>*T*, *c.1360C*>*T*, *c.487G*>*A*, *c.1004C*>*A*, *c.835T*>*A*, *c.383G*>*T*, and *c.202G*>*A*. Figure 4 lists the number of male hemizygotes and female heterozygotes detected for the six more common G6PD mutations. The most common mutation was G6PD Kaiping (*1388G*>*A*), with 278 male hemizygotes and 261 female heterozygotes detected, followed by G6PD Canton (*1376G*>*T*), with 245 male hemizygotes and 234 female heterozygotes detected. In addition, some mutations that are less common in China were detected, such as G6PD Union (*1360C*>*T*), G6PD Mahidol (*487G*>*A*), G6PD Fushan (*1004C*>*A*), G6PD Chinese-1 (*835T*>*A*), G6PD Salerno (*383G*>*T*) and Asahi (*202G*>*A*) (Table 1).

**Table 1:** The number of mutations with fewer carriers and enzyme activity

Genotype	Male hemizygotes		Female heterozygotes	
	Number of carriers	G6PD activity (U/L)	Number of carriers	G6PD activity (U/L)
c.1360C>T	1	149	6	1170.33 ± 559.31
c.487G>A	2	198.00 ± 33.94	1	1199
c.1004C>A	–	–	2	1154.00 ± 121.62
c.383G>T	–	–	1	660
c.202G>A	1	634	–	–
c.835T>A	1	192	–	–

Two male heterozygotes (*c.1388G*>*A*, *c.1376G*>*T*) and 2 male compound heterozygotes (*c.1388G*>*A/c.95A*>*G*, *c.1376G*>*T/c.95A*>*G*) were detected. A total of 17 female homozygotes and 45 female compound heterozygotes were detected (Table 2). Among female homozygotes, the *c.1388G*>*A* mutation was the most common, found in 8 females, followed by the *c.1376G*>*T* mutation, found in 6 females, and the *c.95A*>*G* mutation, found in 3 females. The most common compound mutation detected was *c.1388G*>*A/c.1376G*>*T*, found in a total of 20 individuals (Table 2).

**Table 2:** A total of 17 female homozygotes, 45 female compound heterozygotes, 2 male heterozygotes, and 2 male compound heterozygotes were identified

Genotype	Female compound heterozygotes		Female homozygotes		Male compound heterozygotes		Male heterozygotes	
	Number of carriers	G6PD activity (U/L)	Number of carriers	G6PD activity (U/L)	Number of carriers	G6PD activity (U/L)	Number of carriers	G6PD activity (U/L)
c.1388G>A	–	–	8	292.00 ± 128.20	–	–	1	543.00
c.1376G>T	–	–	6	236.00 ± 65.23	–	–	1	1266.00
c.95A>G	–	–	3	267.33 ± 109.46	–	–	–	–
c.1376G>T/c.1388G>A	20	299.25 ± 294.91	–	–	–	–	–	–
c.1376G>T/c.95A>G	8	342.63 ± 261.84	–	–	1	314	–	–
c.1376G>T/c.871G>A	1	181.00	–	–	–	–	–	–
c.1376G>T/c.1024C>T	2	204.00 ± 104.48	–	–	–	–	–	–
c.1388G>A/c.95A>G	5	450.60 ± 327.21	–	–	1	23	–	–
c.1388G>A/c.392G>T	3	317.67 ± 149.56	–	–	–	–	–	–
c.1388G>A/c.871G>A	1	387.00	–	–	–	–	–	–
c.1388G>A/c.1024C>T	1	514.00	–	–	–	–	–	–
c.392G>T/c.871G>A	1	306.00	–	–	–	–	–	–
c.1024C>T/c.1360C>T	1	1057.00	–	–	–	–	–	–
c.95A>G/c.835T>A	1	222.00	–	–	–	–	–	–
c.1024C>T/c.95A>G	1	220.00	–	–	–	–	–	–
Total	45	338.38 ± 279.98	17	267.88 ± 103.39	2	168.50 ± 205.76	2	904.50 ± 511.24

We calculated the genotype frequencies for the different G6PD mutations. The frequency of female heterozygotes was calculated based on the male allele frequency, and this result was consistent with the detected female heterozygous frequency (Table 3).

**Table 3:** Allele frequencies for different G6PD genotypes

Genotype	Allele frequency	Frequency of female heterozygotes (calculated based on male allele frequencies)	Frequency of female heterozygotes
c.1388G>A	0.026 (278/10751)	0.025	0.027 (261/9457)
c.1376G>T	0.023 (245/10751)	0.022	0.025 (234/9457)
c.95A>G	0.010 (106/10751)	0.010	0.010 (100/9457)
c.871G>A	0.006 (60/10751)	0.006	0.005 (43/9457)
c.392G>T	0.003 (27/10751)	0.003	0.005 (46/9457)
c.1024C>T	0.002 (24/10751)	0.002	0.003 (31/9457)

Comparison of the frequency of female heterozygotes with the frequency of female heterozygotes calculated from the male allele frequency

### Sequencing results

The results of DNA sequencing showed 6 missense mutations in this study population, including *c.187G*>*A*, *c.703C*>*T*, *c.551C*>*T*, *c.517T*>*C*, *c.1438A*>*T*, and *c.946G*>*A* (Table 4). *c.551C*>*T* was first reported in Madhya Pradesh, India, and was named G6PD Dindori after the region [15], but it has not previously been reported in China. *c.703C*>*T* was reported in Guangxi, China, and was named G6PD Nanning after the region [16]. *c.187G*>*A* was found in Taiwan, China, and was named G6PD Pingtung after the region [17]. *c.517T*>*C*, named G6PD Nankang, has been previously reported in China and Malaysia [18, 19]. *c.1438A*>*T* and *c.946G*>*A* are novel mutations found in this study and have not been reported previously.

**Table 4:** Missense mutations obtained from DNA sequencing results

cDNA change	Protein change	Case No	Genotype	Sex	G6PD activity (U/L)	Reported
c.1438A>T	p.Ile480Phe	1	Hemizygote	Male	943	This study
c.946G>A	p.Gly316Ser	2	Hemizygote	Male	1047	This study
c.551C>T	p.Ser184Phe	3	Homozygote	Female	20	India
c.703C>T	p.Leu235Phe	4	Homozygote	Female	1043	Guangxi China
		5	Hemizygote	Male	1195	
c.187G>A	p.Glu63Lys	6	Hemizygote	Male	1260	Taiwan China
c.517T>C	p.Phe173Leu	7	Heterozygote	Female	887	China, Malaysia
		8	Heterozygote	Female	519	
		9	Heterozygote	Female	920	
		10	Hemizygote	Male	222	
		11	Hemizygote	Male	255	
		12	Hemizygote	Male	334	

### Correlation analysis of G6PD mutations and G6PD enzyme activity

**Fig. 5:**
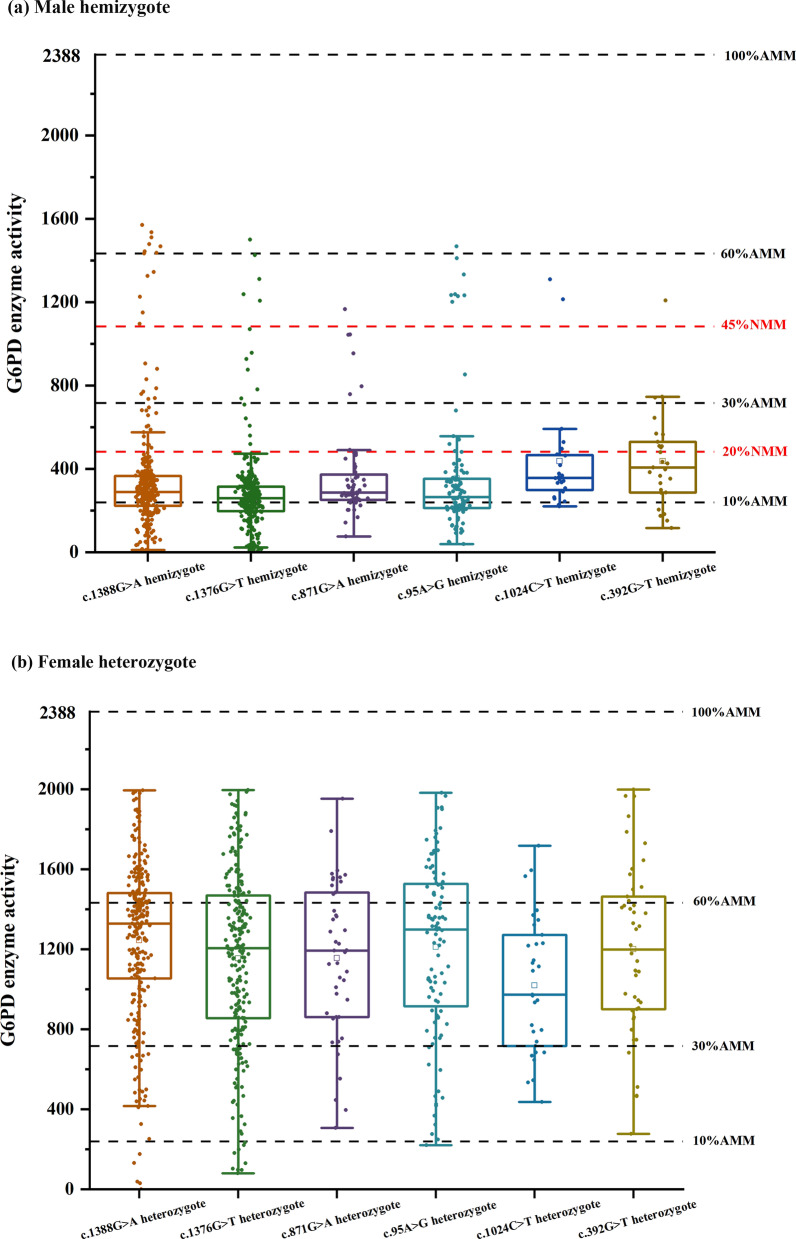
Box plot of the enzyme activity of females heterozygous for each mutation and males hemizygous for each mutation. (The black four reference lines in the panel for male hemizygotes indicate 10%, 30%, 60% and 100% of the AMM (10% AMM, 30% AMM, 60% AMM and 100% AMM, respectively). The two red lines represent the reference lines for class **A** and class **B** of the new WHO classification. The four reference lines in the panel for female heterozygotes indicate 10% AMM, 30% AMM, 60% AMM and 100% AMM)

Six prevalent G6PD mutations were selected for correlation analysis with G6PD activity. Among males hemizygous for the *c.1376G*>*T*, *c.1388G*>*A*, and *c.95A*>*G* mutations, nearly half of the patients had enzyme activity values that were < 10% of the AMM. In males hemizygous for the *c.871G*>*A*, *c.392G*>*T*, and *c.1024C*>*T* mutations, enzyme activity values were mostly between 10 and 60% of the AMM. Nearly all males with G6PD mutations had enzyme activity values less than 30% of the AMM (an enzyme activity value that is 30% of the AMM is an acceptable level of G6PD activity for primaquine administration) [13, 14]. On the other hand, most female heterozygotes had enzyme activity values that were > 10% of the AMM, and their enzyme activity was more widely distributed. This result indicates that the enzyme activity value of male hemizygotes is lower, which means that the clinical manifestations caused by G6PD mutations may be more severe (Fig. 5).

According to the new WHO classification of G6PD variants in 2022, it is recommended that the percent activity value should be calculated from the genotypically normal male median (NMM) value, not from the AMM value, considering the overlap of most variants across the 10% threshold [5]. We calculated that the median number of male individuals with normal genotypes was 2408 U/L. The WHO Class A median of G6PD activity was < 481.6 U/L (G6PD activity < 20%), and the WHO Class B median of G6PD activity was < 1083.6 U/L (G6PD activity < 45%). According to the new classification of variants for hemizygous males [5], the median values of the male hemizygous population for the six common prevalent mutations were all below 20%NMM (Fig. 5).

Comparing the enzyme activities between participants carrying the different missense mutations, we found significant differences in the enzyme activities (Table 5). The findings suggested that there were differences in enzyme activities among the different mutations.

**Table 5:** The 6 missense mutations identified in this study

Genotype	Male hemizygotes		*P* value	Female heterozygotes		*P* value
	Number of carriers	G6PD activity (U/L)		Number of carriers	G6PD activity (U/L)	
c.1376G>T	245	286.62 ± 213.44	*P* < 0.05	261	1154.41 ± 439.38	*P* < 0.05
c.1388G>A	278	350.17 ± 274.73		234	1244.97 ± 294.27	
c.871G>A	60	363.15 ± 220.72		43	1155.31 ± 382.75	
c.95A>G	106	352.90 ± 300.69		100	1211.03 ± 433.13	
c.392G>T	27	436.81 ± 227.95		46	1199.77 ± 422.45	
c.1024C>T	24	436.01 ± 271.56		31	1019.32 ± 338.31	

We divided enzyme activities into different ranges and compared the mutation detection rates between each group. Table 6 shows that as enzyme activity increased, the detection rate of mutations decreased in both females and males, indicating that the probability of carrying mutations decreased with increasing G6PD enzyme activity. With increased enzyme activity, the probability of carrying mutations was significantly reduced in males and slightly decreased in females.

**Table 6:** The connection between G6PD genotyping assays and G6PD phenotyping assays

G6PD enzyme activity (U/L)	Female positive rate (%)	Male positive rate (%)
0–300	95.4	96.9
301–600	97.0	100.0
601–900	96.6	87.5
901–1200	91.2	65.0
1201–1500	59.8	32.9
1501–1800	34.4	7.40*
1801–2000	15.0	–

“*” represents the calculated male enzyme activity ranging from 1501 to 1600 U/L

## Discussion

G6PD deficiency has long been recognized as a common inherited haematological disorder in southern China, and screening for and the prevention of G6PD deficiency are essential for public health [11, 12]. At present, the primary method of screening for G6PD deficiency in the clinic is a quantitative assay of G6PD activity. The measurement of G6PD activity plays an important role in neonatal screening for and the differential diagnosis of neonatal jaundice. In female heterozygous patients, the range of enzyme activities is broad due to the random inactivation of the X chromosome, so detecting G6PD deficiency by measuring enzyme activity may lead to a missed diagnosis [20]. Therefore, genetic testing is crucial to increase the female heterozygote detection rate. According to the reference range of G6PD enzyme activity provided by the kit, genotyping was performed for all samples with G6PD enzyme activity below 1300 U/L. In addition, the male samples with G6PD enzyme activity between 1300 and 1600 U/L (120% of 1300 U/L as the boundary) and females with enzyme activity between 1300 and 2000 U/L (150% of 1300 U/L as the boundary) were also genotyped to identify more genotype-positive people.

Different mutations of the G6PD gene cause different levels of enzyme deficiency, with classes assigned to various degrees of deficiency and disease manifestations. Because there are significant differences in enzyme activity between carriers of different variants, genotyping is essential because carriers of different genotypes may have different clinical manifestations and abilities to resist oxidative stress damage. In China, a malaria-free country, primaquine is rarely used. However, no G6PD activity level has been established as an indication for the use of common oxidative drugs in China, and there are few related studies. The oxidative medications prescribed to patients with G6PD deficiency should be appropriately selected on the basis of enzyme activity and mutation sites, and when to use them should be decided with care. In regard to female homozygotes, female compound heterozygotes, and male compound heterozygotes, more care should be taken when using drugs. Among individuals with low enzyme activity (0–300 U/L), there are individuals with negative genotyping results and negative sequencing results, which suggests that there may be some unknown intron- or promoter-related regulatory mechanism [21].

G6PD deficiency is remarkable for its genetic diversity. The three most frequently detected mutations in this study were *c.1376G*>*T*, *c.1388G*>*A*, and *c.95A*>*G*, which is consistent with epidemiological survey results in China [12]. According to the new classification, the six common prevalent mutations can be classified as Class A. The median values of men with normal genotypes calculated from our data do not represent the true NMM because mutations may be present in samples with enzyme activity ≥ 1600 U/L in males even if the probability of detecting the mutation is very small in this case. Therefore, the practical operation of calculating AMM for large-scale population screening may be more convenient. Our finding is consistent with the obvious regional or ethnic specificity of G6PD mutations. Because of globalization and immigration, we detected mutations that are more commonly found in other regions. We identified two G6PD Mahidol (*487G*>*A*) male hemizygotes and one G6PD Mahidol (*487G*>*A*) female heterozygote. G6PD Mahidol (*487G*>*A*) is more common in the Thai population [22]. We found one G6PD Salerno (*383G*>*T*) female heterozygote and one Asahi (*202G*>*A*) male hemizygote. In Indonesia, *c.383G*>*T* is more prevalent, and *c.202G*>*A* is more prevalent in Africa [23]. Two male compound heterozygotes (*c.1388G*>*A/c.95A*>*G*, *c.1376G*>*T/c.95A*>*G*) were found. This phenomenon indicates the presence of mutations at two different sites on one X chromosome in males or may suggest Klinefelter syndrome [24]. A *c.1388G*>*A* male heterozygote and a *c.1376G*>*T* male heterozygote were found, both of whom had undergone female allogeneic haematopoietic stem cell transplantation (HSCT). HSCT involves the transfer of the donor haematopoietic stem cells (HSCs) to the recipient [25]. When the donor is a female heterozygous for a G6PD mutation, heterozygosity may be detected in a male patient after transplantation. F Pilo et al. evaluated the safety of HSCT with G6PD-deficient donors [26]. The safety and efficacy of transplantation outcomes with 19 G6PD-deficient donors and 82 G6PD-normal donors were compared. There was no difference between the two groups in any safety parameter or transplant outcome. The authors concluded that a G6PD-deficient but otherwise healthy volunteer could be selected as an HSC donor. However, trimethoprim-sulfamethoxazole (cotrimoxazole), frequently used to prevent *Pneumocystis carinii* prophylaxis in HSCT recipients, is a major oxidative drug [27]; therefore, G6PD deficiency is also a concern in the population undergoing allogeneic HSCT, as this may involve some medication problems.

Among the six missense mutations obtained from DNA sequencing results, 4 missense mutations (*c.187G*>*A*, *c.703C*>*T*, *c.551C*>*T*, *c.517T* > *C*) were reported as pathogenic. *c.1438A*>*T* and *c.946G*>*A* have not been previously reported. However, these new mutations still require attention. However, further studies are needed to determine the pathogenicity and pathogenesis of these two variants.

## Conclusion

In summary, we conducted a comprehensive screening of G6PD deficiency in a population from Guangzhou and found two previously unreported mutations. The detection of compound heterozygotes and heterozygotes in the male population could provide relevant genetic counselling. The mutation spectrum of G6PD deficiency was elucidated, which could be helpful for genetic counselling and the prevention of this disease in Guangzhou.

## Abbreviations

G6PD: Glucose-6-phosphate dehydrogenase
NADPH: Nicotinamide Adenine Dinucleotide Phosphate
CNSHA: Chronic Nonspherical Haemolytic Anaemia
WHO: World Health Organization
AMM: Adjusted Male Median
NMM: Normal Male Median
MPCR: Multiplex Polymerase Chain Reaction
PCR: Polymerase Chain Reaction
MALDI-TOFMS: Matrix-Assisted Laser Desorption Ionization Time of Flight Mass Spectrometry
HSCT: Haematopoietic Stem Cell Transplantation
